# HIV Drug Resistance Early Warning Indicators in Namibia with Updated World Health Organization Guidance

**DOI:** 10.1371/journal.pone.0100539

**Published:** 2014-07-02

**Authors:** Anna Jonas, Victor Sumbi, Samson Mwinga, Michael DeKlerk, Francina Tjituka, Scott Penney, Michael R. Jordan, Tiruneh Desta, Alice M. Tang, Steven Y. Hong

**Affiliations:** 1 Directorate of Special Programmes, Republic of Namibia Ministry of Health and Social Services, Windhoek, Namibia; 2 Strengthening Pharmaceutical Systems, Management Sciences for Health, Windhoek, Namibia; 3 Department of Public Health and Community Medicine, Tufts University School of Medicine, Boston, Massachusetts, United States of America; 4 Division of Geographic Medicine and Infectious Diseases, Tufts Medical Center, Boston, Massachusetts, United States of America; 5 World Health Organization Namibia, Klein Windhoek, Namibia; Fundacion Huesped, Argentina

## Abstract

**Background:**

In response to concerns about the emergence of HIV drug resistance (HIVDR), the World Health Organization (WHO) has developed a comprehensive set of early warning indicators (EWIs) to monitor HIV drug resistance and good programme practice at antiretroviral therapy (ART) sites.

**Methods:**

In 2012, Namibia utilized the updated WHO EWI guidance and abstracted data from adult and pediatric patients from 50 ART sites for the following EWIs: 1. *On-time Pill Pick-up*, *2. Retention in Care*, *3. Pharmacy Stock-outs*, *4. Dispensing Practices*, and *5. Virological Suppression*.

**Results:**

Data for EWIs one through four were abstracted and validated. *EWI 5 – Virological Suppression* was not included due to poor data entry at many sites. *On-time Pill Pick-up* national estimate was 87.9% (87.2–88.7) of patients picking up pills on time for adults and 90.0% (88.9–90.9) picking up pills on time for pediatrics. *Retention in Care* national estimate was 82% of patients retained on ART after 12 months for adults and 83% for pediatrics. *Pharmacy Stock-outs* national estimate was 99% of months without a stock-out for adults and 97% for pediatrics. *Dispensing Practices* national estimate was 0.01% (0.003–0.064) of patients dispensed mono- or dual-therapy for adults and 0.25% (0.092–0.653) for pediatrics.

**Conclusions:**

The successful 2012 EWI exercise provides Namibia a solid evidence base, which can be used to make national statements about programmatic functioning and possible HIVDR. This evidence base will serve to contextualize results from Namibia's surveys of HIVDR, which involves genotype testing. EWI abstraction has prompted the national program and its counterparts to engage sites in dialogue regarding the need to strengthen adherence and retention of patients on ART. The EWI collection process and EWI results will serve to optimize patient care and support Namibia in making evidence-based recommendations and take action to minimize the emergence of preventable HIVDR.

## Introduction

### Background

In recent years, the rapid scale up of antiretroviral therapy (ART) for treatment of HIV infection in resource-limited countries has been highly successful resulting in 9.7 million people receiving ART in low- middle-income countries as of December 2012 [1]. The public health approach to scaling up ART in resource-limited settings involves the use of standardized and simplified treatment regimens that are consistent with international standards, and appropriate to local circumstances. Because of the high mutation rate and high replication rate of HIV, the chronic nature of HIV infection and the need for lifelong treatment, the emergence of some drug resistance is inevitable in populations taking ART [2]–[3].

In response to countries concerns about the emergence of HIV drug resistance (HIVDR), the World Health Organization (WHO) has developed a comprehensive HIVDR surveillance and monitoring strategy based on public health principles. The updated 2012 global HIVDR surveillance and monitoring strategy contains 5 key elements: *1. Monitoring of Early Warning Indicators (EWI) of HIVDR*, *2. Surveillance of transmitted drug resistance (TDR) in recently infected populations*, *3. Surveillance of HIVDR in populations initiating ART*, *4. Surveillance of acquired HIVDR in populations on ART*, and *5. Surveillance of HIVDR in children <18 months of age* [4].

### HIVDR Early Warning Indicators

The purpose of routine monitoring of HIVDR EWIs is to assess the extent to which ART sites and programmes are functioning by monitoring factors at individual ART sites known to create situations favourable to the emergence of HIVDR. The monitoring of EWIs provides the context for interpreting the results from surveys of HIVDR. Specifically, EWI results permit the timely identification of ART sites not achieving a globally suggested standard target, which supports tailoring of appropriate interventions that can potentially optimize care and treatment and reduce the risk of population-level HIVDR emergence. Drug resistance will not necessarily result immediately if an indicator shows non-optimal performance; however, achieving the best possible performance as measured by these indicators will help to minimize preventable HIVDR.

In 2012, WHO updated its 2010 EWI guidance by conducting a critical review of the available medical literature and the multiple challenges observed with data collection and reporting. EWI definitions were simplified and harmonized with other monitoring and evaluation frameworks and processes, including those of the Global Aids Response Progress Reporting (GARPR) and the United States President's Emergency Plan for AIDS Relief (PEPFAR) [4]. The number of core indicators was reduced to four: on-time pill pick-up, dispensing practices, drug supply continuity and clinic retention at 12 months. A fifth indicator, viral load suppression at 12 months, was recommended to be monitored only at sites where viral load testing was routinely performed on all patients 12 months after therapy initiation. EWI targets were adjusted to take into account new scientific evidence on optimal programme management and performance.

### HIV in Namibia

Namibia is a resource-limited country in sub-Saharan Africa that has been severely affected by the HIV epidemic. In Namibia, there are approximately 200,000 people living with HIV in a population of 2.1 million [5]. Among 15–49 year olds, approximately 18.2% are infected with HIV-1. [unpublished data] The epidemic is predominantly spread via heterosexual contact, and prevalence estimates vary by region with up to 37.7% infected with HIV-1 in the most heavily affected areas in the north. [unpublished data]

### ART rollout

**Figure 1:**
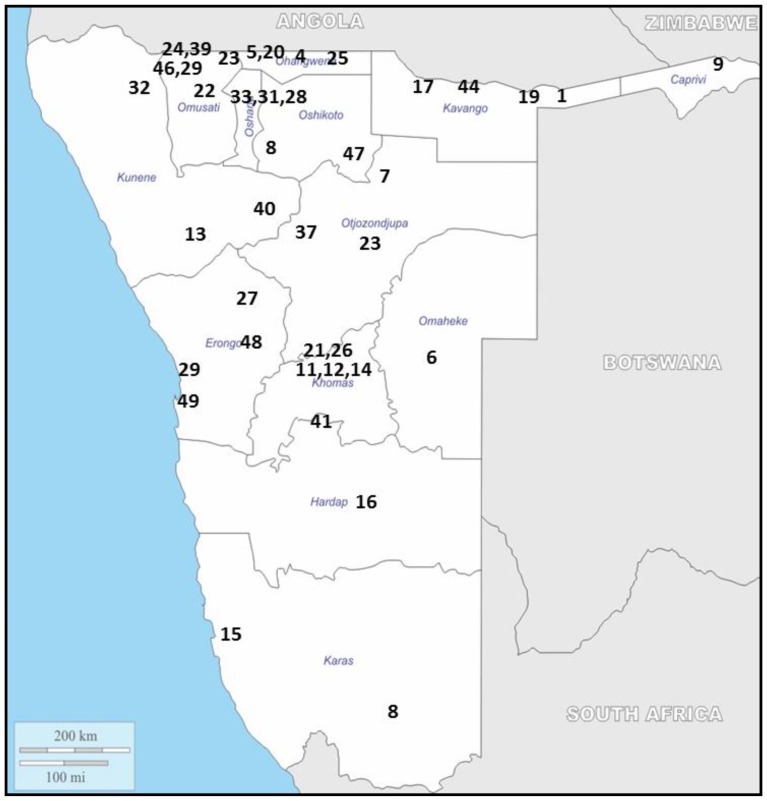
Geographic Location of ART Sites. This figure illustrates the public ART sites located in Namibia. The map is adapted from: http://d-maps.com/carte.php?num_car=4824&lang=en.

ART has been available in Namibia's private sector since 1997 and in the public sector since 2003. At 84%, Namibia has one of the highest ART coverage rates in Sub-Saharan Africa with 107,154 eligible patients on ART as of March 2013. At present, ART is available at all 40 public hospitals and at an additional 111 satellite/outreach service points, as well as 30 Integrated Management of Adolescent and Adult Illness (IMAI) sites. [unpublished data] Of these sites, the national ART program considers 44 to be main ART sites. Main sites dispense ART independently and to patients at IMAI and satellite/outreach sites. (Figure 1)

In the public sector, ART is provided free of charge following a population-based model of care with one primary first-line regimen and three alternate first-line regimens consisting of two nucleoside reverse transcriptase inhibitors (NRTI) combined with a non-nucleoside reverse transcriptase inhibitor (NNRTI). The recommended second-line regimen consists of 2 NRTIs with a ritonavir-boosted protease inhibitor (PI). ART initiation is based on WHO clinical staging and/or CD4 cell count ≤350 cells/mm^3^. All public ART sites have access to first- and second-line ART regimens. At all public ART sites, viral load testing is performed six months after ART initiation and targeted viral load testing is performed to confirm clinical or immunological failure beyond six months [6]. With support from Management Sciences for Health (MSH) Namibia, a standardized pharmacy record system, the Electronic Dispensing Tool (EDT), is used to dispense all ART. In the private sector, ART is provided utilizing an individual model of care with ART regimens selected based on results of drug resistance testing. (16% of patients on ART in Namibia)

### Early Warning Indicators in Namibia

In 2009, Namibia piloted five EWIs at nine ART sites [7]: 1) *ART prescribing practices*, 2) *Patients lost to follow-up (LTFU) at 12 months*, 3) *Patient retention on first-line ART at 12 months*, 4) *On-time ARV drug pick-up*, and 5) *ARV drug-supply continuity*. Records supported monitoring of three of these five EWIs. Nine of nine (100%) sites met the target of 100% initiated on appropriate first-line regimens. Eight of nine (89%) sites met the target of ≤20% LTFU. Six of nine (67%) sites met the target of 0% switched to a second-line regimen. In 2010, Namibia scaled-up these same three EWIs from nine to 33 ART sites [8]. Twenty-two of 33 (67%) sites met the target of 100% initiated on appropriate first-line regimens. Seventeen of 33 (52%) sites met the target of ≤20% LTFU. Fifteen of 33 (45%) sites met the target of 0% switched to a second-line regimen. EWI monitoring directly resulted in public health action to optimize the quality of care, specifically the strengthening of ART record systems, engagement of ART sites, and operational research for improved adherence assessment and improved ART patient defaulter tracing.

## Methods

### Early Warning Indicators selection

Based on discussion with WHO consultants and review of pertinent record-keeping systems, the Namibia HIVDR technical working group (TWG) determined to abstract the following EWIs for abstraction in 2012 with the corresponding WHO-recommended targets summarized in Table 1: *On-time pill pick-up*, *Retention in care*, *Pharmacy stock-outs*, *Dispensing practices*, and *Virological Suppression (Namibia-specific definition due to routine viral load testing done at 6 months in Namibia).*

**Table 1:** Selected 2012 WHO Early Warning Indicator Definitions (Numerator/Denominator) and Targets.

Early Warning Indicator	Definitions (Numerator/Denominator)	Targets#
**On-time pill pick-up**	Numerator: number of patients picking up their ART on time* at first drug pick-up after a defined baseline pick-up date.	Red: <80%
	Denominator: number of patients who picked up drugs on or after the designated EWI sample start date.§	Amber: 80–90%
		Green: >90%
**Retention in care**	Numerator: number of adults or children who are still alive and on ART 12 months after initiating treatment.	Red: <75% retained after 12 months of ART
	Denominator: total number of adults or children who initiated ART who were expected to achieve 12-month outcomes within the reporting period, including those who have died since starting therapy, those who have stopped therapy, and those recorded as lost to follow-up† at month 12.∞	Amber: 75–85% retained after 12 months of ART
		Green: >85% retained after 12 months of ART
**Pharmacy stock-outs**	Numerator: number of months in the designated year in which there were no stock-out‡ days of any (adult or pediatric) ARV drug routinely used at the site.	Red: <100% of a 12-month period with no stock-outs
	Denominator: 12 months.	Green: 100% of a 12-month period with no stock-outs
**Dispensing practices**	Numerator: number of patients (adults or children) who pick up form the pharmacy, a regimen consisting of one or two ARVs.	Red: >0% dispensing of mono- or dual therapy
	Denominator: number of patients (adults or children) picking up ART on or after the designated EWI sample start date. Sampling continues until the full sample size is reached.	Green: 0% dispensing of mono- or dual therapy
**Virological suppression at 6 months** ∧	Numerator: number of patients receiving ART and a viral load at the site after the first 6 months of ART whose viral load is<1000 copies/mL.	Targets to be determined by WHO
	Denominator: consecutive ART starters from 1 January, 2010 until 31 December 2010 and have viral load results after 6-months available (between 5–12 months from ART initiation).	

ART – Antiretroviral therapy.

ARV – Antiretrovirals.

*On-time pill pick-up: Pick up pills no more than two days late on their first pick-up after a baseline pick-up.

§ EWI sample start date: The date designated as the start of the sampling. The sample start date is fixed by the HIVDR Working Group.

† Lost to follow-up: Patients who had not returned to the pharmacy or clinic ≤90 days after the last ART run-out date during the 12-months after the date of ART initiation were classified as LTFU. Stopping therapy without restarting was classified as not LTFU if the patient continued to attend clinic appointments.

∞ Transfers of care to another site were excluded from the denominator.

‡ Stock-out: Any occurrence of zero stock of a routinely-used ARV drug at the site at which the patient routinely picks up ARVs.

# Adult and pediatric targets are the same. Targets for *Virological Suppression at 6-months* have not been determined by WHO.

∧ Due to routine data collection of viral load at 6 months, Namibia chose to monitor *Virological suppression at 6 months* instead of the WHO recommendation of 12 months.

Table adapted from WHO HIV Drug Resistance EWI guidance report [4].

EWI performance was rated according to WHO recommended scorecards. (Table 1) The scorecards utilize three classifications: red (poor performance, below desired level), amber (fair performance, not yet at desired level), and green (excellent performance, achieving desired level). Also, the scorecards allow for a “grey” classification if a site does not monitor a specific EWI or a “white” classification if an indicator is not reported according to national regulations [4].

EWIs are monitored separately for adult and pediatric populations. Recommended targets are identical for adult and pediatric populations except for the indicator assessing desirable rates of virological suppression.

### Ethics statement

Ethical review was not required as this data was public health surveillance data abstracted from existing routinely collected ministry of health medical records. Only anonymized data were abstracted from the medical records for public health surveillance purposes. Names, dates of birth, addresses, and unique patient identifier numbers were not abstracted from records. After discussion with the Tufts Medical Center institutional review board, it was determined that because this was routine public health de-identified data analyzed within the Ministry of Health and Social Services in Namibia, no formal written waiver was necessary. The data used for this study was obtained from and analyzed by the Ministry of Health and Social Services (MoHSS) of Namibia.

### Site selection and data abstraction

EWIs are designed to be collected routinely from all sites within a country, or a large number of representative sites. All 44 main ART delivery sites and 6 outreach sites that had disaggregated EDT data were chosen for EWI abstraction in 2012 including sites that participated in EWI abstraction in 2010. Data for ART outreach sites without disaggregated EDT data were included in this year's EWI abstraction exercise within the main ART sites. EWI data abstraction was conducted centrally in August 2012 by a data abstraction team formed by the TWG in collaboration with the WHO and the MoHSS. The team consisted of 4 members trained on the WHO methodology of EWI abstraction. Data for *On-time pill pick-up*, *Retention in care*, and *Dispensing Practices* were downloaded from the national EDT database through automatic queries into MS Excel and calculated according to WHO guidance. Data for *Pharmacy stock-outs* were calculated from monthly ART site reporting. Data for *Virological suppression at 6 months* were downloaded centrally from the national electronic patient medical system (ePMS) database into MS Excel.

### Data quality assessment

Data quality assessments were implemented throughout the EWI process. Three elements of data quality were considered in the assessments: data reliability, data completeness, and data consistency [9]. Data reliability, which is an assessment of the quality of the abstraction, was assessed by confirming 10% of the centrally-queried data to the existing data in the EDT. Data completeness was assessed from the centrally-queried data; and sites with a large percentage of data missing were removed from EWI analyses (1 site). Finally, assessment of data consistency was initially performed during the pilot of EWIs [7] and the most optimal source for each variable was determined. EDT data were considered the gold standard for pharmacy pick-up dates and ART regimens dispensed, while ePMS and paper records (Patient Care Booklets) were considered the gold standard for information about patient status such as dates of transfer in and transfer out, dates of death, and dates of stop. Therefore, EDT data for patients who had incomplete pill pick-ups were validated and corrected by comparing records in ePMS and Patient Care Booklets, looking for dates of transfer out, death or ART stop. Centrally-queried EDT data for patients who had inappropriate ART regimens at start or at 12 months were validated and corrected with the site-specific EDT system to ensure accuracy of the queries. Validation with ePMS and paper medical records was performed by the individual ART sites that were trained on EWI methodology at a national EWI conference.

### Sample size

In order to make the results generalizable to the patient population at the ART site, the sampling strategy was based on calculating a minimum sample size for each indicator at each site, based on the number of eligible patients for each EWI. For *On-time pill pick-up* and *Dispensing practices*, the number of eligible patients at each site to be sampled was the number of patients who were “active” on ART at the time of the sample start date (1 January, 2012). WHO recommends data abstraction on a minimum number of consecutive patients following the sample size criteria below to provide a 95% CI of ±7%. However, data abstraction was oversampled by 20% to account for potential censoring of patients; therefore the true confidence intervals are <±7%. All sites began abstraction from the sample start date, and abstracted data until appropriate sample size for each site was reached; regardless of how many months it took to reach the appropriate sample size. Sample sizes for sites were based on the numbers of patients at each participating ART site meeting the eligibility definition of patients to be represented for each EWI according to WHO guidance [4]. For *Retention in care*, a census of all patients initiating ART in the 12 months of 2010 was taken (consistent with GARPR/PEPFAR). For *Virological suppression at 6 months*, a census of all patients starting ART in 2010 with 6-month viral load data available was obtained.

### Calculation of national estimates

**Figure 2:**
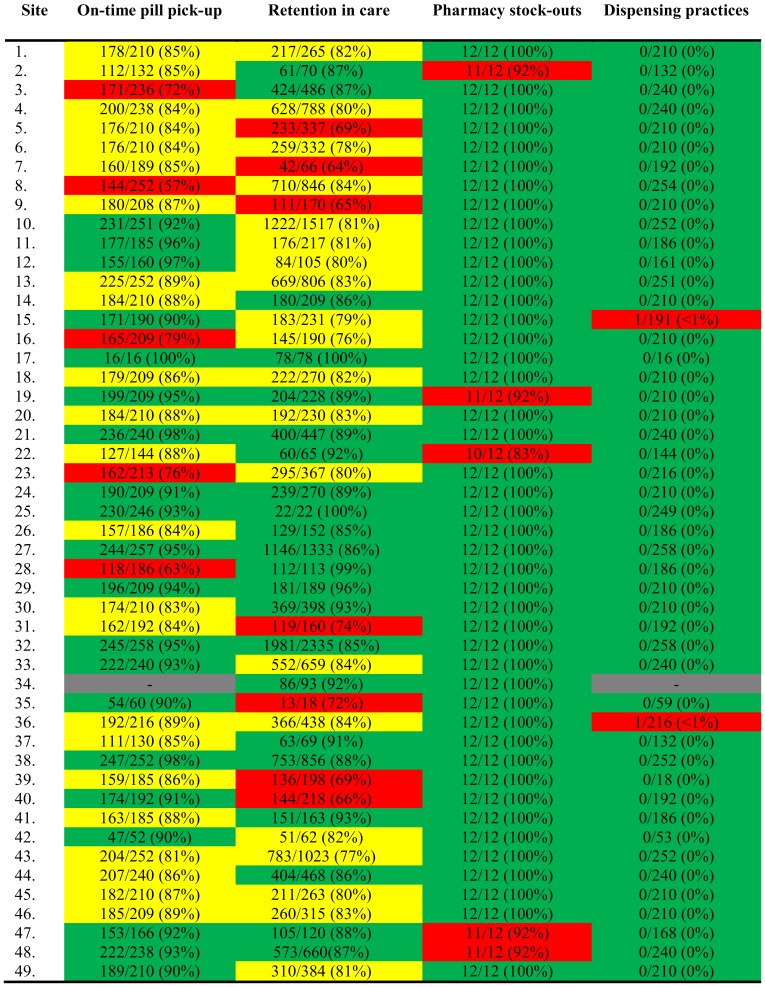
Adult Site-specific EWI Results. EWI – Early Warning Indicator. Green indicates sites that achieved excellent performance, desired target level. Yellow indicates sites that achieved fair performance, progressing towards desired target level. Red indicates poor performance, below desired target level. Gray indicates that data was not available from that site.

**Figure 3:**
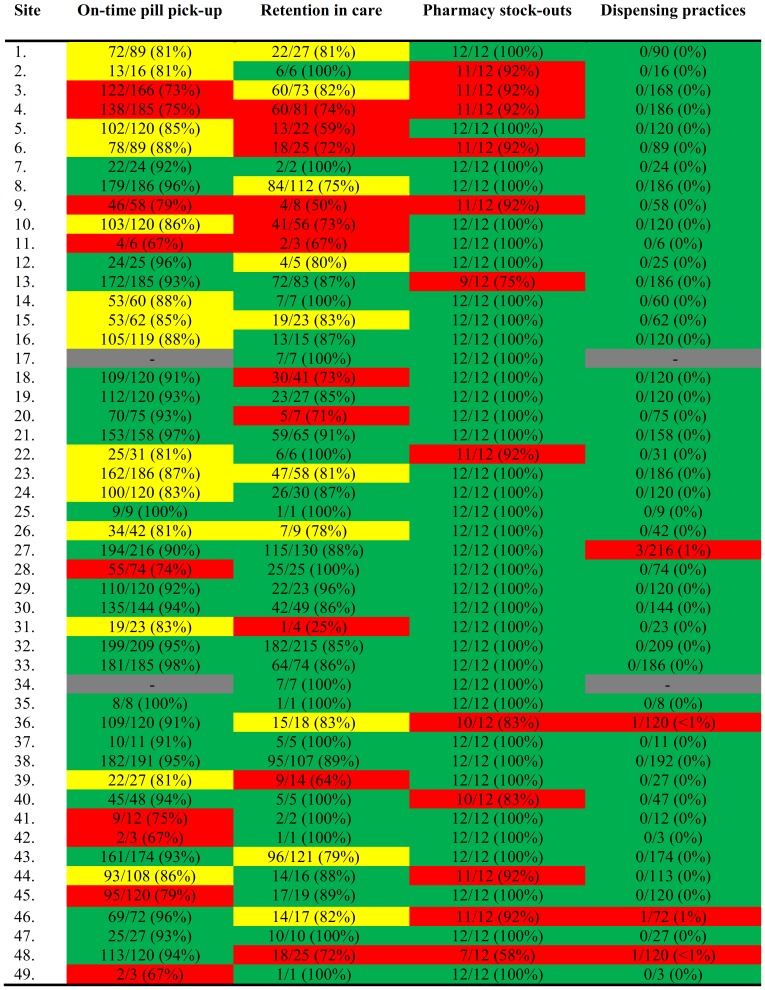
Pediatric EWI Site-specific Results. EWI – Early Warning Indicator. Green indicates sites that achieved excellent performance, desired target level. Yellow indicates sites that achieved fair performance, progressing towards desired target level. Red indicates poor performance, below desired target level. Gray indicates that data was not available from that site.

The national estimates for adult and pediatric EWIs were calculated by summing the numerators and denominators for each EWI found in Figures 2 and 3, respectively, to get a cumulative percentage of all ART sites. (Table 2) The national statistics are representative of Namibia's public ART sites because data for all outreach sites not listed in this report were included within the main sites. This means that all patients receiving ART in the public sector in Namibia were included in the sample frame.

**Table 2:** National EWI Summary Report.

Early Warning Indicator	EWI Target for all sites (time period)	Number adult of sites meeting EWI target (% of sites meeting target)	Number pediatric of sites meeting EWI target (% of sites meeting target)N = X ART sites	National Adult Estimates% (CI)	National Pediatric Estimates% (CI)
**On-time pill pick-up**	Green: >90%	Green 20/48 (42%)	Green 23/47 (49%)	87.9% (87.2–88.7)*	90.0% (88.9–90.9)*
	Amber: 80–90%	Amber 23/48 (48%)	Amber 15/47 (32%)		
	Red: <80%	Red 5/48 (10%)	Red 9/47 (19%)		
	(1 Jan 2012-)				
**Retention in care**	Green: >85%	Green 22/49 (45%)	Green 28/49 (57%)	15,957/19,299 (82%)	1,399/1,688 (83%)
	Amber: 75–85%	Amber 20/49 (41%)	Amber 10/49 (21%)		
	Red: <75%	Red 7/49 (14%)	Red 11/49(22%)		
	(1 Jan 2010–31 Dec 2010)				
**Pharmacy stock-outs**	Green: 100%	Green 44/49 (90%)	Amber 37/49 (76%)	582/588 (99%)	568/588 (97%)
	Red: <100%	Red 5/49 (10%)	Red 12/49 (24%)		
	(1 April 2012–31 Mar 2013)				
**Dispensing practices**	Green: 0%	Green 46/48 (96%)	Green 42/47 (89%)	0.01% (0.003–0.064)*	0.25% (0.092–0.653)*
	Red: >0%	Red 2/48 (4%)	Red 5/47 (11%)		
	(1 Jan 2012-)				

EWI – Early Warning Indicator.

*National estimates were weighted by the number of active patients and proportion of patients sampled from each ART site.

The confidence intervals for EWIs 1 and 4 were weighted by number of active patients and proportion of total patients sampled at each ART site. Confidence intervals were calculated separately for adults and pediatrics populations attending the same clinic. *Retention in care* and *Pharmacy stock-out* confidence intervals were not calculated because retention is measured as a census of all patients and stock outs are reports of actual drug shortages.

## Results

Namibia abstracted data on four EWIs for both adults and pediatrics: *On-time pill pick-up*, *Retention in care*, *Pharmacy stock-outs*, and *Dispensing practices*. Data from 28,909 adults and 6,086 pediatric patients were abstracted and analyzed. Site-specific EWI results for adults are presented in Figure 2 and for pediatrics in Figure 3. The national EWI summary for adults and pediatrics is presented in Table 2. Data collected for *Virological suppression at 6 months* was determined to be unreliable due to site-level data entry errors.

### On-time pill pick-up

For adults, 42% of sites achieved “excellent” performance (≥90%) for *On-time pill pick-up*, 48% of sites had “fair” performance (80–90%), and 10% of sites had “poor” performance (<80%). The rates from all adult sites ranged from 57% to 100%. For pediatrics, 49% of the sites achieved “excellent” performance (≥90%), 32% of sites had “fair” performance (80–90%), and 19% of sites had “poor” performance (<80%). The rates from all pediatric sites ranged from 67% to 100%. The national estimate for *On-time pill pick-up* was 87.9% (87.2–88.7) of patients picking up pills on time for adults and 90.0% (88.9–90.9) picking up pills on time for pediatrics.

### Retention in care

For adults, 45% of the sites achieved “excellent” performance (≥85%) for *Retention in care*, 41% of sites had “fair” performance (75–85%), and 14% of sites had “poor” performance (<75%). The retention rates from the adult sites ranged from 64% to 100%. For pediatric sites, 57% of sites achieved “excellent” performance (≥85%), 21% of sites had “fair” performance (75–85%), and 22% of sites had “poor” performance (<75%). The retention rates at the pediatric sites ranged from 25% to 100%. The national estimate for *Retention in care* is 82% of patients retained on ART after 12 months for adults and 83% for pediatrics.

### Pharmacy stock-outs

For adults, 90% of sites achieved “excellent” performance with 100% of months without a pharmacy stock-out and 10% were classified as “poor” performance with <100% of months without a pharmacy stock-out. For pediatrics, 76% of sites achieved “excellent” performance and 24% of sites were classified as “poor” performance. The national estimate for *Pharmacy stock-outs* is 99% of months without a stock-out for adults and 97% for pediatrics.

### Dispensing practices

For adult sites, 96% achieved “excellent” performance with 0% of patients dispensed mono- or dual therapy, and 4% were classified as “poor” performance with >0% of patients mono- or dual-therapy. For pediatric sites, 91% of achieved “excellent” performance and 9% were classified as “poor” performance. Only 8 of 14,008 patients were dispensed dual therapy. There was no dispensing of mono-therapy. The national estimates for *ARV dispensing practices* are 0.01% (0.003–0.064) of patients dispensed mono- or dual-therapy for adults and 0.25% (0.092–0.653) for pediatrics.

### Virological suppression at 6 months

It was discovered that in many sites, ART clerks were not entering VL data into the ePMS that had the results “target not detected” (undetectable). Therefore, VL suppression data are expected to be underestimates. So the decision was made to report these data as “grey” for “data not available”. It was also discovered that a proportion of VL were being conducted before ART start at some sites.

## Discussion

This paper presents the first published HIVDR EWIs using the new 2012 WHO guidance and the first published pediatric EWI data for Namibia. Namibia successfully abstracted data on four WHO recommended EWIs and scaled-up monitoring to 50 ART sites throughout the country from the previous 33 ART sites in 2010: *On-time pill pick-up*, *Retention in care*, *Pharmacy stock-outs*, and *Dispensing practices*. The 50 ART sites which include data from main and outreach/satellite sites represent the public ART sites throughout the country. For the first time in Namibia, existing medical and pharmacy records (ePMS, EDT, and Patient care booklets) allowed for accurate monitoring of four EWIs. Also pediatric data were abstracted for the first time. Accurate monitoring of *Virological suppression at 6 months* was not accomplished due to systematic data entry errors at site-level. For the previous EWI exercise in 2010, accurate data abstraction was not possible from existing medical and pharmacy records to report *On-time pill pick-up*, *Pharmacy stock-outs*, or pediatric EWI data. The 2010 EWI exercise resulted in important modifications to the data abstraction tool to make this EWI data abstraction possible [8].

*On-time pill pick-up* is an important measure of patient adherence that is associated with LTFU [10], HIVDR [11]–[13] virological failure [14]–[17], and increased mortality [18]–[20]. In Namibia, over 40% of adult and pediatric sites achieved the target of ≥90% *On-time pill pick-up* rates. In previously published EWI data in other African settings, only 15% of the 321 adult sites monitoring *On-time pill pick-up* achieved their target of ≥90% [21]. Similarly, published data from Cameroon revealed 0% of their sites achieving ≥90% on-time pill pick-up rates [22]. However, comparisons are limited because Namibia utilized the new WHO definition of *On-time pill pick-up*. With these data, Namibia plans to design operational research to investigate the sites performing poorly for site-level factors contributing to poor population adherence.

In Namibia, less than half of all adult ART sites achieved the target of ≥85% retention at 12 months with pediatric sites performing only slightly better. According to Fox and Rosen [23], sub-Saharan Africa reported an average of 80.2% retention at 12 months with LTFU being the highest contributing factor to attrition. In Namibia, data suggest a significant proportion of patients are not retained in care due to LTFU or transferring out to other ART clinics without informing their previous clinic. A recent paper in Malawi [24] found that out of 2,183 LTFU patients who were traced and alive, 1,226 (56%) were reported to be still taking ART from the original clinic or another clinic. Out of the 1,226, 293 (24%) patients reported treatment gaps. Therefore, it is possible that the low observed retention rates may be underestimates. Nevertheless, a substantial proportion of these patients may be at high risk for experiencing treatment interruptions and developing HIVDR [25]–[26]. According to Brinkhof et al [27], patients that are not retained have increased mortality, 40% of LTFU patients, with most of the mortality occurring during the first 6 months of disengagement with care. Namibia's broad range of retention rates between ART sites suggest there may be factors at site-level that are influencing retention. Efforts should be made to investigate reasons for disengagement from care in order to strengthen and standardize existing defaulter tracing mechanisms. Acting upon EWI data, Namibia has initiated operational research to examine reasons for LTFU and factors associated with LTFU. Additionally, an intervention study is planned to investigate the effect of defaulter tracing.

In previous EWI exercises in Namibia, it was not possible to monitor *Pharmacy stock-outs* or drug supply continuity due to inaccurate stock records [7]–[8]. However, modifications to the stock reporting system resulted in available data abstraction for this important EWI. In 2012, very few adult sites and a small number of pediatric sites had ART stock outs in Namibia. According to Bennett et al, in Africa 63% percent of 537 adult sites achieved the target of 100% of 12 months with no stock-outs in a 2012 aggregate analysis [21]. Additionally, Billong et al [22] reported 45% of 38 adult sites and Sigaloff et al [28] documented 75% of 12 adult sites reaching the WHO target. *Pharmacy stock-outs* is an important EWI to report because it is strongly associated with HIVDR and virological suppression [29]. The reasons for stock-outs in Namibia were poor inventory management practices, storage space constraints, and short-dated ARVs. Based on these data Namibia plans to strengthen supervision by regional pharmacists in order to ensure proper drug forecasting, procurement, and supply distribution. Long-term solutions for lack of storage space at certain ART sites are being investigated. Also, communication between the ART logistics pharmacist and the Central Medical Stores are being strengthened in order to determine appropriate ARV stock levels.

In Namibia, very few sites were found to have inappropriate prescribing of adult or pediatric regimens. In other African countries the reported percent of sites meeting the WHO target were, 74% of 907 sites [21], 90% of 40 sites [22], 88% of 81 sites [30], and 85% of 13 sites [28]. However, comparisons are limited by the change in the WHO definition for inappropriate regimens. Using the updated WHO definition (mono- or dual-therapy), very few ART sites in Namibia did not meet the WHO target for inappropriate dispensing. Furthermore, the small percentage of sites that did not meet the WHO target had <1% of patients dispensed mono or dual therapy. In investigating these cases of mono- or dual-therapy, most were found to be transfers in from the private sector which were continued on these regimens by the ART clinic staff. Based on these data, Namibia plans to use clinical mentors to investigate patients on inappropriate regimens. In addition, plans are underway to engage the private sector in order to determine prescribing practices.

Due to availability of data, Namibia chose to monitor *Virological suppression at 6 months* instead of the WHO EWI recommendation of monitoring viral load at 12 months [4]. In the data analysis phase, it was discovered that data clerks at many ART sites were not correctly entering in undetectable viral loads into the ePMS. Therefore, the viral load suppression rates are likely gross underestimates. Although this exercise resulted in inaccurate estimates of virological suppression, lessons learned will be used to educate lab data entry clerks on the proper procedures for entering undetectable viral loads so that this important indicator can be accurately monitored in future years.

Since Namibia sampled representatively from all public ART sites in the country, a national estimate of each EWI was calculated. A nationally representative estimate can be used to monitor trends over time and compare the functioning of the ART program with internationally accepted standards. Also, individual ART sites can be compared to the national standard in order to determine sites underperforming and need further investigation. In addition, sites performing above national standards can be investigated for best practices which can then be applied to other ART sites. Comparisons were also made between adult and pediatric ART sites, which did not find any notable difference.

One important limitation of this EWI exercise is the inability to collect reliable data on *Virological suppression at 6 months*. Strengthening of ART data quality, as mentioned above, is an important factor for future monitoring. An additional limitation is these data were not disaggregated into outreach/satellite and IMAI sites for analysis, even though data for these sites were included in the main sites. Moving forward, Namibia plans to disaggregate EWI data into the IMAI and outreach/satellite sites from the main sites so that programmatic functioning can be assessed at every level of care. Also, as decentralisation of ART services continues, data quality will be strengthened at IMAI and outreach sites. Finally, the limitation to engage the private sector in EWI exercises prevents the ability to make broader statements about ART delivery and HIVDR in Namibia. Also, many patients in the public sector transfer in from the private sector and the lack of communication may lead to inaccurate data. Efforts are being made to include the private sector in future EWI exercises.

The successful 2012 EWI exercise built upon two previous rounds of EWIs [7]–[8] provides Namibia a solid evidence base, which can be used to make national statements about programmatic functioning in the context of HIVDR and related factors. This evidence base will serve to contextualize results from Namibia's surveys of HIVDR, which involve HIV genotype testing. Currently, analysis of data from national surveys of acquired and transmitted HIVDR is ongoing; nationally representative surveillance of pre-treatment and acquired HIVDR is planned.

The EWI abstraction process has mobilized the national ART program and its partners to institute minor adjustments in existing databases, which will facilitate abstraction of WHO recommended EWIs in the future (viral load suppression), and yield a more accurate assessment of overall programmatic functioning. Importantly, three successful rounds of EWI monitoring have highlighted the potential for HIVDR emergence in Namibia due to sites not optimizing adherence and retention of patients. These data have prompted the national program and its counterparts to engage sites in dialogue regarding the need to strengthen adherence and retention of patients on ART. The EWI collection process and EWI results will serve to optimize patient care and support Namibia in making evidence-based recommendations and take action to minimize the emergence of preventable HIVDR.

EWIs in Namibia have been integrated into the routine Monitoring and Evaluation activities of the MoHSS, thereby ensuring sustainability into the future. EWIs are routinely monitored at site level and reported as an ongoing activity with continuous validation along with annual national reporting.
